# Eigenvalue Adjustment-Based STAP in Airborne MIMO Radar Under Limited Snapshots

**DOI:** 10.3390/s26051508

**Published:** 2026-02-27

**Authors:** Chao Xu, Qizhen Feng, Zhao Wang, Dingding Li, Di Song

**Affiliations:** 1 Civil Aviation Flight University of China, Deyang 618307, China; best_xc@cafuc.edu.cn (C.X.); fy00014138@cafuc.edu.cn (Q.F.); wangzhao@cafuc.edu.cn (Z.W.); lidingding@cafuc.edu.cn (D.L.); 2 School of Physics and Electronic Engineering, Nanyang Normal University, Nanyang 473061, China; 3 School of Electronic and Optical Engineering, Nanjing University of Science and Technology, Nanjing 210094, China

**Keywords:** covariance matrix estimation, multiple-input multiple-output, space-time adaptive processing, limited snapshots

## Abstract

The covariance matrix performs a vital role for space-time adaptive processing (STAP) in airborne multiple-input multiple-output (MIMO) radar. As is known, the clutter-plus-noise covariance matrix (CPNCM), reflecting the statistical characteristics of radar echo, is a key component for MIMO-STAP. Commonly, an ideal CPNCM is impossible to obtain, and it must be estimated with sufficient snapshots. According to the RMB rule, MIMO-STAP requires many snapshots since MIMO radar has a high degree-of-freedom (DoF) due to its orthogonal transmit waveform. However, this is hard to satisfy in practice. This paper develops a novel covariance matrix estimation method under limited snapshots in airborne MIMO-STAP radar. Motivated by the random matrix theory, the proposed method enhances the CPNCM estimation by noise and clutter sample eigenvalues adjustment (EA). Concretely, the sample eigenvalues of noise are adjusted as noise power, and the ones of clutter are adjusted through minimizing the radar output power. Then, with the sample eigenvectors and adjusted sample eigenvalues, an effective CPNCM is formulated, and EA-MIMO-STAP is implemented reliably. Multiple experiments demonstrate that EA-MIMO-STAP has superior performance and robustness.

## 1. Introduction

Since the seminal work by Bekkerman in 2006 [1], multiple-input multiple-output (MIMO) radar has garnered wide interest. Broadly speaking, MIMO radars have two primary types: distributed MIMO [2,3,4] and collocated MIMO [5,6,7]. This paper specifically concentrates on the latter. The fundamental concept of MIMO radar entails a radar system that transmits multiple (typically orthogonal) waveforms via a transmit array and subsequently processes the corresponding echoes at the receiver. In comparison with conventional phased-array radars, MIMO radar offers the distinct capability of effectively forming larger virtual arrays with relatively smaller physical antenna dimensions. To a certain extent, this innovative approach addresses the limitations faced by airborne radar antennas, such as constrained aperture and weight due to the carrier platform, and enhances angular resolution. In general, the remarkable benefits of a large virtual array aperture and an extended pulse accumulation time enable MIMO radar to substantially elevate target detection performance.

Space-time adaptive processing (STAP) is a highly effective technology for detecting targets in airborne radar systems. Since Brennan and Reed’s pioneering work in 1973 [8], STAP has garnered wide attention. STAP exhibits exceptional prowess in clutter suppression and target detection, owing to its capacity to jointly leverage spatial and temporal information [9,10,11]. When STAP is applied to a MIMO radar system, it becomes even more advantageous for detecting slow-moving targets amidst a strong clutter environment. This synergy enables the attainment of superior ground moving target indication performance. Consequently, MIMO-STAP has become hot topic in airborne radars.

In the implementation of MIMO-STAP, the statistical characteristics for clutter and noise must be obtained, i.e., the clutter-plus-noise covariance matrix (CPNCM), which is a vital component in MIMO-STAP weight vector design. With the ideal CPNCM, MIMO-STAP optimizes the signal-to-interference-plus-noise ratio (SINR). Nevertheless, it is not possible to get the ideal CPNCM practically because of the work environmental variation of airborne radar [10]. Conventionally, the CPNCM is often approximated through the sample covariance matrix (SCM) formulated with snapshots. When numbers of snapshots are limited, performance deterioration will occur in MIMO-STAP. In particular, the renowned Reed–Mallet–Brennan (RMB) rule [12] offers a clear theoretical framework for determining the necessary amount of snapshots, i.e., to keep the performance loss below 3 dB, the amount of snapshots needs to be twice the system DoF. Unfortunately, in practical scenarios, satisfying the RMB rule is often challenging.

### 1.1. Related Works

Several approaches are proposed to tackle this issue. Reduced-dimension (RD)-MIMO-STAP can significantly decrease the system DoF with a transformation matrix, then reduce the requirement on snapshots by the RMB rule [13,14,15,16,17,18]. Nonetheless, the decrease in system DoF unavoidably leads to performance loss. Knowledge-aided (KA)-MIMO-STAP introduces extra information into CPNCM estimation, such as radar and platform parameters, the clutter environment, and terrain and mapping information, thereby improving the CPNCM’s performance under limited snapshots [19,20,21,22,23,24]. Nonetheless, the accuracy of the extra information decides KA-MIMO-STAP’s performance directly. In practical environments, the information about the clutter environment and platforms is commonly inaccurate, causing KA-MIMO-STAP performance degradation. Sparse-recovery (SR)-MIMO-STAP can exploit a few snapshots to recover the clutter profile and then obtain an accurate estimation of the CPNCM [25,26,27,28,29,30]. Nevertheless, SR-MIMO-STAP spends too much time recovering the CPNCM, and this is hard to use for real-time environments. Therefore, it is imperative to apply other measures to enhance MIMO-STAP under limited snapshots.

As is known, the CPNCM can be separated into eigenvalues and eigenvectors. With the advancement of random matrix theory originating from quantum mechanics, its research focus has predominantly centered on consistent estimations of eigenvalues/eigenvectors under limited observations [31,32,33,34,35,36]. This development offers a fresh perspective for enhancing the estimation of the CPNCM when snapshots are scarce, given that the CPNCM can be decomposed into eigenvalues and eigenvectors. Stein illustrated that the SCM could be significantly enhanced if sample eigenvalues were effectively adjusted [37,38]. Furthermore, a multitude of studies have substantiated the efficacy of this covariance matrix estimation strategy. The experiments have also unequivocally shown that a covariance matrix with eigenvalue adjustment (EA) outperforms the SCM [39,40,41,42]. Along with these studies, this paper precisely adopts the idea to estimate the inverse CPNCM through EA. Notably, the spiked model within random matrix theory, i.e., a low-rank perturbation of an identity matrix [43], garners substantial attention because of its alignment with clutter-plus-noise. Aiming toward this framework, a plethora of studies [43,44,45,46] have indicated that consistent estimations of eigenvalues can be achieved with a few snapshots. This theoretical foundation has been successfully leveraged to enhance the performance of radar and communication signal processing. Given these insights, it is highly promising to refine the CPNCM estimation by employing EA in conjunction with a spiked framework.

### 1.2. Our Contributions

This paper introduces a groundbreaking MIMO-STAP approach, namely EA-MIMO-STAP, which adjusts sample eigenvalues under limited snapshots and is well-suited for addressing MIMO-STAP issues. Note that the eigenvalues of the CPNCM can be divided into clutter eigenvalues and noise eigenvalues. The former are significantly bigger than the latter. Moreover, the noise eigenvalues constitute a substantial portion, and they are equivalent to the variance. In contrast, the clutter eigenvalues account for a minor part, and they are decided according to clutter variance. This clutter-plus-noise covariance model is in perfect alignment with the spiked model. In this model, consistent estimation of eigenvalues is achieved according to [43,44,45,46]. This consistency holds not only as the observation number is infinite while the observation dimension remains fixed but also when the observation number rises to infinity with the dimension under the same rate. In MIMO-STAP, noise eigenvalues are adjusted with the power, and clutter ones are adjusted through minimizing radar output power and consistently estimating the spiked eigenvalues. With eigenvalue adjustment for noise and clutter, an effective estimate of the inverse CPNCM can be obtained. Subsequently, the MIMO-STAP weight vector can be generated.

The key contributions are summarized as follows:

- Different from the existing MIMO-STAP methods, the method in this paper first exploits the outstanding outcomes of consistent estimation of the isolated eigenvalues of the spiked covariance model in random matrix theory for use in MIMO-STAP for CPNCM estimation, though this scheme appears naturally.
- This paper divides the eigenvalues into clutter eigenvalues and noise eigenvalues and adjusts them. In other words, we design the eigenvalues according to the specific property in MIMO-STAP, and this processing procedure is different from the existing eigenvalue adjustment methods.
- Compared with the existing MIMO-STAP, EA-MIMO-STAP exhibits superior performance when dealing with limited snapshots. Additionally, the experiments demonstrate robustness given the existence of noise power estimation errors, array gain/phase errors, and internal clutter motion.

The remaining structure of this paper is arranged as follows. In Section 2, the signal model applicable to airborne radar, as well as the fundamental principles in MIMO-STAP, are presented. Section 3 delves into a detailed description of the proposed EA-MIMO-STAP algorithm. Subsequently, Section 4 describes a series of numerical experiments, and ultimately, Section 5 summarizes the conclusions.

Notation: $I_{N}$ stands for the N×N identity matrix. ⊗ denotes the Kronecker product. ${\left(\cdot \right)}^{H}$ stands for the Hermitian transpose. ℂ stands for the sets of complex values. E(⋅) denotes statistical expectation.

## 2. Background

### 2.1. Signal Model

**Figure 1:**
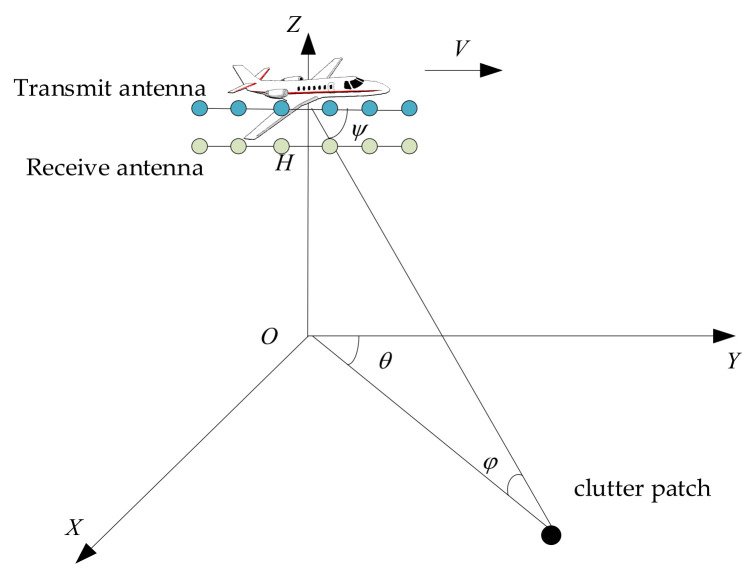
Airborne MIMO radar.

A side-looking airborne MIMO radar is demonstrated in Figure 1. In the radar system, there are E transmitting and K receiving uniform linear array elements spaced by $\sigma_{T}$ and $\sigma_{R}$, respectively. The carrier frequency is denoted by $f_{c}$. Here H and V represent altitude and speed. φ, θ, and ϕ represent the elevation, azimuth, and cone angle. Under one coherent processing interval, assume that there are M emitted pulses with pulse repetition interval $T_{\mathrm{PRI}}$. Under this assumption without range ambiguity, the radar echo $x_{i}\in {\mathbb{C}}^{EMK}$ in the i-th range bin after match filtering can be given as

(1) $$x_{i}=s+c_{i}+n,$$

where $s\in {\mathbb{C}}^{EMK}$ stands for the target, $n\in {\mathbb{C}}^{EMK}$ denotes noise with mean 0 and variance $\varepsilon_{n}$ in the receiver, and $c_{i}\in {\mathbb{C}}^{EMK}$ stands for clutter echo,

(2) $$\begin{array}{ll} c_{i} & =\sum_{p=1}^{T_{p}}\chi_{i,p}b\left(f_{t}\left(\phi_{i,p}\right),f_{d}\left(\phi_{i,p}\right),f_{r}\left(\phi_{i,p}\right)\right)\\ & =\sum_{p=1}^{M_{p}}\chi_{i,p}b_{T}\left(f_{t}\left(\phi_{i,p}\right)\right)⊗b_{D}\left(f_{d}\left(\phi_{i,p}\right)\right)⊗b_{R}\left(f_{r}\left(\phi_{i,p}\right)\right) \end{array}.$$

In (2), $T_{p}$ represents the clutter patch number, $\chi_{i,p}$ represents the amplitude of the clutter patch, and $b\left(f_{t}\left(\phi_{i,p}\right),f_{d}\left(\phi_{i,p}\right),f_{r}\left(\phi_{i,p}\right)\right)$ denotes the steering vector of clutter. $b_{T}\left(f_{t}\left(\phi_{i,p}\right)\right)\in {\mathbb{C}}^{E}$, $b_{D}\left(f_{d}\left(\phi_{i,p}\right)\right)\in {\mathbb{C}}^{M}$ and $b_{R}\left(f_{r}\left(\phi_{i,p}\right)\right)\in {\mathbb{C}}^{K}$ denote the transmit, Doppler and receive steering vectors,

(3) $$\begin{array}{cc} b_{T}\left(f_{t}\left(\phi_{i,p}\right)\right) & ={\left[1,e^{j2\pi f_{t}\left(\phi_{i,p}\right)},\cdots ,e^{j2\pi f_{t}\left(\phi_{i,p}\right)\left(E-1\right)}\right]}^{T}\\ b_{D}\left(f_{d}\left(\phi_{i,p}\right)\right) & ={\left[1,e^{j2\pi f_{d}\left(\phi_{i,p}\right)},\cdots ,e^{j2\pi f_{d}\left(\phi_{i,p}\right)\left(M-1\right)}\right]}^{T}\\ b_{R}\left(f_{r}\left(\phi_{i,p}\right)\right) & ={\left[1,e^{j2\pi f_{r}\left(\phi_{i,p}\right)},\cdots ,e^{j2\pi f_{r}\left(\phi_{i,p}\right)\left(K-1\right)}\right]}^{T} \end{array},$$

where $f_{t}\left(\phi_{i,p}\right)$, $f_{d}\left(\phi_{i,p}\right)$ and $f_{r}\left(\phi_{i,p}\right)$ respectively stand for the transmit, Doppler and receive frequencies,

(4) $$\begin{array}{cc} f_{t}\left(\phi_{i,p}\right) & =\sigma_{T}\cos\left(\phi_{i,p}\right)/\lambda \\ f_{d}\left(\phi_{i,p}\right) & =2VT_{PRI}\cos\left(\phi_{i,p}\right)/\lambda \\ f_{r}\left(\phi_{i,p}\right) & =\sigma_{R}\cos\left(\phi_{i,p}\right)/\lambda \end{array},$$

where $\lambda =f_{c}/c$ denotes the radar wavelength and c denotes the speed of light.

Then, the MIMO-STAP problem can be described as

(5) $$\left\{\begin{array}{ll} \min & \rho \left(w\right)\\ s.t. & w^{H}b\left(f_{t}^{s},f_{d}^{s},f_{r}^{s}\right)=1 \end{array}\right.,$$

where $\rho \left(w\right)=w^{H}R_{c+n}w$ stands for the radar output power, $b\left(f_{t}^{s},f_{d}^{s},f_{r}^{s}\right)\in {\mathbb{C}}^{EMK}$ represents the target steering vector, and $R_{c+n}\in {\mathbb{C}}^{EMK\times EMK}$ denotes the CPNCM. Commonly, the clutter is independent of noise; then

(6) $$\begin{array}{ll} R_{c+n} & =E\left[\left(c_{i}+n\right){\left(c_{i}+n\right)}^{H}\right]\\ & =E\left[c_{i}c_{i}^{H}+{\mathrm{nn}}^{H}\right]\\ & =\sum_{p=1}^{T_{p}}\begin{array}{l} {\left|\chi_{i,p}\right|}^{2}b\left(f_{t}\left(\phi_{i,p}\right),f_{d}\left(\phi_{i,p}\right),f_{r}\left(\phi_{i,p}\right)\right)\\ b^{H}\left(f_{t}\left(\phi_{i,p}\right),f_{d}\left(\phi_{i,p}\right),f_{r}\left(\phi_{i,p}\right)\right) \end{array}+\varepsilon_{n}I_{EMK} \end{array}.$$

With the CPNCM $R_{c+n}$ in (6), the optimal MIMO-STAP weight $w\in {\mathbb{C}}^{EMK}$ can be calculated through solving (5) as follows:

(7) $$w_{opt}=\frac{R_{c+n}^{-1}b\left(f_{t}^{s},f_{d}^{s},f_{r}^{s}\right)}{b^{H}\left(f_{t}^{s},f_{d}^{s},f_{r}^{s}\right)R_{c+n}^{-1}b\left(f_{t}^{s},f_{d}^{s},f_{r}^{s}\right)}.$$

Then, the minimized radar output power can be derived as

(8) $$\begin{array}{ll} \rho_{\min}\left(w\right) & ={\rho \left(w\right)|}_{w=w_{opt}}\\ & =\frac{1}{b^{H}\left(f_{t}^{s},f_{d}^{s},f_{r}^{s}\right)R_{c+n}^{-1}b\left(f_{t}^{s},f_{d}^{s},f_{r}^{s}\right)} \end{array}.$$

### 2.2. MIMO-STAP with Limited Snapshots

As shown in (7), optimal MIMO-STAP can be implemented if an accurate inverse CPNCM $R_{c+n}^{-1}$ can be obtained. Nonetheless, it is hard in practice to know the ideal CPNCM $R_{c+n}$, which is generally replaced by the SCM ${\hat{R}}_{c+n}$ with limited snapshots, i.e.,

(9) $${\hat{R}}_{c+n}=\sum_{i=1}^{D}x_{i}x_{i}^{H},$$

where D denotes the number of snapshots. In this case, with (9), the optimal MIMO-STAP from (7) can be rewritten as

(10) $$\hat{w}=\frac{{\hat{R}}_{c+n}^{-1}b\left(f_{t}^{s},f_{d}^{s},f_{r}^{s}\right)}{b^{H}\left(f_{t}^{s},f_{d}^{s},f_{r}^{s}\right){\hat{R}}_{c+n}^{-1}b\left(f_{t}^{s},f_{d}^{s},f_{r}^{s}\right)},$$

where $\hat{w}$ denotes the full-dimension (FD)-MIMO-STAP weight. Therefore, the radar output power from (10) is derived as

(11) $$\begin{array}{ll} \rho \left(\hat{w}\right) & ={\rho \left(w\right)|}_{w=\hat{w}}\\ & =\frac{b^{H}\left(f_{t}^{s},f_{d}^{s},f_{r}^{s}\right){\hat{R}}_{c+n}^{-1}R_{c+n}{\hat{R}}_{c+n}^{-1}b\left(f_{t}^{s},f_{d}^{s},f_{r}^{s}\right)}{{\left[b^{H}\left(f_{t}^{s},f_{d}^{s},f_{r}^{s}\right){\hat{R}}_{c+n}^{-1}b\left(f_{t}^{s},f_{d}^{s},f_{r}^{s}\right)\right]}^{2}} \end{array}.$$

In (11), it is found that if ${\hat{R}}_{c+n}^{-1}$ is equal to $R_{c+n}^{-1}$, MIMO-STAP can realize optimal processing. For limited snapshots, the difference between ${\hat{R}}_{c+n}^{-1}$ and $R_{c+n}^{-1}$ is large, and $\rho \left(\hat{w}\right)$ also gets large. Then, MIMO-STAP’s performance will become poor.

Traditionally, some methods have been proposed to overcome the significant performance loss with limited snapshots, such as RD-MIMO-STAP, KA-MIMO-STAP, and SR-MIMO-STAP. These methods have their specific applicable scopes. However, the work environments of airborne radars vary quickly, and this makes these methods hard to apply. Therefore, it is of paramount and urgent importance to find alternative strategies to optimize the performance of MIMO-STAP when confronted with a scarcity of snapshots.

### 2.3. Eigenvalue Adjustment-Based MIMO-STAP

Notice that the purpose of MIMO-STAP is to derive an excellent $R_{c+n}^{-1}$ estimation according to the available ${\hat{R}}_{c+n}^{-1}$ under limited snapshots. Motivated by [39,40,41,42], this work improves the SCM through adjusting the sample eigenvalues, and superior performance can be obtained. To this end, we consider adjusting the eigenvalues of ${\hat{R}}_{c+n}^{-1}$ to improve the estimation of ${\hat{R}}_{c+n}^{-1}$.

Here, the decomposition of $R_{c+n}$ is analyzed:

(12) $$R_{c+n}=\sum_{l=1}^{EMK}\alpha_{l}u_{l}u_{l}^{H},$$

where $\alpha_{l}$ represents the l-th eigenvalue with corresponding eigenvector $u_{l}$ and $\alpha_{1}\geq \alpha_{2}\geq \cdots \geq \alpha_{L}\gg \alpha_{L+1}=\cdots \alpha_{EMK}$. Because the noise eigenvalue of the CPNCM is equal to the noise power, i.e., $\alpha_{L+1}=\cdots \alpha_{EMK}=\varepsilon_{n}$, we have

(13) $$\begin{array}{ll} R_{c+n} & =\sum_{l=1}^{L}\alpha_{l}u_{l}u_{l}^{H}+\sum_{l=L+1}^{EMK}\varepsilon_{n}u_{l}u_{l}^{H}\\ & =\sum_{l=1}^{L}\left(\alpha_{l}-1\right)u_{l}u_{l}^{H}+\sum_{l=1}^{EMK}\varepsilon_{n}u_{l}u_{l}^{H}\\ & =\varepsilon_{n}\left(\sum_{l=1}^{L}{\overset{⌢}{\alpha }}_{l}u_{l}u_{l}^{H}+I_{EMK}\right) \end{array},$$

where L represents the clutter rank, which could be derived as in [47], and ${\overset{⌢}{\alpha }}_{l}=\left(\alpha_{l}-1\right)/\varepsilon_{n}$. Notice that the largest L eigenvalues are clutter eigenvalues, while the others represent noise eigenvalues. MIMO-STAP will become worse as L increases [2].

It is well-known that the number of DoFs EMK far exceeds the clutter rank L, and the clutter eigenvalue is much larger than that of the noise. This eigenvalue model aligns perfectly with the spiked structure model [43,44,45,46]. This specific model has garnered extensive research attention due to its distinctive statistical properties. For limited snapshots, consistent estimation of the isolated eigenvalues has been successfully studied [43,44,45,46]. With this accomplishment, $R_{c+n}^{-1}$ can be precisely estimated through an eigenvalue adjustment algorithm. Consequently, this method enables a distinct improvement in MIMO-STAP performance.

## 3. MIMO-STAP by Eigenvalue Adjustment

**Figure 2:**
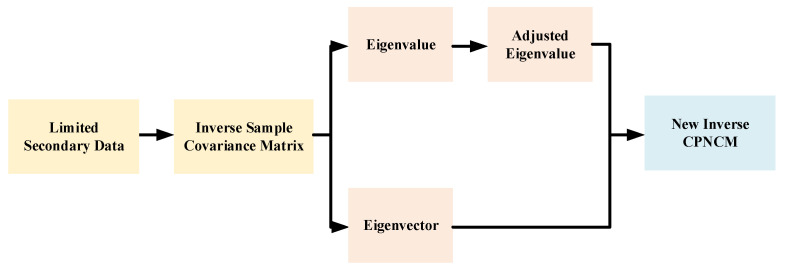
The basic processing procedure of EA-MIMO-STAP.

This section presents an MIMO-STAP approach achieved by eigenvalue adjustment, namely EA-MIMO-STAP. The basic processing procedure is shown in Figure 2. Initially, the problem of EA-MIMO-STAP can be established within the framework of the spiked covariance model. Subsequently, we proceed to derive the asymptotic deterministic equivalence and identify the optimal solution. Finally, we obtain an effective estimation of the EA-MIMO-STAP weight vector.

### 3.1. EA-MIMO-STAP Within the Spiked Framework

In this paper, we mainly would like to adjust the eigenvalues of ${\hat{R}}_{c+n}^{-1}$ and then realize minimization of the radar output power $\rho \left(\hat{w}\right)$ in (11). To do so, we need to rewrite ${\hat{R}}_{c+n}^{-1}$ and $R_{c+n}$ in (11) in the form of eigen-decomposition like (13).

As we know, ${\hat{R}}_{c+n}^{-1}$ and ${\hat{R}}_{c+n}$ have the same eigenvectors, and their eigenvalues are reciprocals of each other. Then, matrix ${\hat{R}}_{c+n}$ can be eigen-decomposed as

(14) $${\hat{R}}_{c+n}=\sum_{l=1}^{EMK}\beta_{l}d_{l}d_{l}^{H}=\sum_{l=1}^{EMK}\varepsilon_{n}{\overset{⌢}{\beta }}_{l}d_{l}d_{l}^{H},$$

where $\beta_{l}$ $\left(\beta_{1}\geq \cdots \geq \beta_{EMK}\right)$ stands for the l-th eigenvalue of ${\hat{R}}_{c+n}$ corresponding to eigenvector $d_{l}$ and $\beta_{l}=\varepsilon_{n}{\overset{⌢}{\beta }}_{l}$. Then

(15) $${\hat{R}}_{c+n}^{-1}=\sum_{l=1}^{EMK}\frac{1}{\beta_{l}}d_{l}d_{l}^{H}=\sum_{l=1}^{EMK}\frac{1}{\varepsilon_{n}{\overset{⌢}{\beta }}_{l}}d_{l}d_{l}^{H}.$$

For eigenvalue adjustment, we should adjust the value $\beta_{l}$ or ${\overset{⌢}{\beta }}_{l}$ and keep $d_{l}$, which is given as

(16) $${\bar{R}}_{c+n}^{-1}=\sum_{l=1}^{EMK}\frac{\gamma_{l}}{\varepsilon_{n}}d_{l}d_{l}^{H},$$

where ${\bar{R}}_{c+n}^{-1}$ is the designed inverse CPNCM with eigenvalues $\gamma_{l}/\varepsilon_{n}$. Then, if the eigenvalues $\gamma_{l}/\varepsilon_{n}$ or $\gamma_{l}$ are optimally adjusted, MIMO-STAP’s performance can be effectively enhanced.

According to the spike model, the eigenvalues of ${\bar{R}}_{c+n}^{-1}$ should also obey the spike covariance model: the noise-associated eigenvalues should be the same and equal to the reciprocal of noise power, i.e., $\gamma_{L+1}=\cdots =\gamma_{EMK}=1$. Therefore, we can rewrite ${\bar{R}}_{c+n}^{-1}$ as

(17) $$\begin{array}{ll} {\bar{R}}_{c+n}^{-1} & =\sum_{l=1}^{EMK}\frac{\gamma_{l}}{\varepsilon_{n}}d_{l}d_{l}^{H}\\ & =\sum_{l=1}^{L}\frac{\gamma_{l}}{\varepsilon_{n}}d_{l}d_{l}^{H}+\sum_{l=L+1}^{EMK}\frac{1}{\varepsilon_{n}}d_{l}d_{l}^{H}\\ & =\left(\sum_{l=1}^{L}{\overset{⌢}{\gamma }}_{l}d_{l}d_{l}^{H}+I_{EMK}\right)/\varepsilon_{n} \end{array}.$$

In (17), the values ${\overset{⌢}{\gamma }}_{l}=\left(\gamma_{l}-1\right)$ $\left(l=1,\cdots ,L\right)$ need to be effectively adjusted for a good estimation of ${\bar{R}}_{c+n}^{-1}$. Then, let the eigenvalues ${\overset{⌢}{\gamma }}_{l}$ $\left(l=1,\cdots ,L\right)$ be arranged as a vector, $\overset{⌢}{\gamma }={\left[{\overset{⌢}{\gamma }}_{1},\cdots ,{\overset{⌢}{\gamma }}_{L}\right]}^{T}$. Therefore, the renewed MIMO-STAP $\bar{w}$ is re-expressed as

(18) $$\bar{w}\left(\overset{⌢}{\gamma }\right)=\frac{{\bar{R}}_{c+n}^{-1}\left(\overset{⌢}{\gamma }\right)b\left(f_{t}^{s},f_{d}^{s},f_{r}^{s}\right)}{b^{H}\left(f_{t}^{s},f_{d}^{s},f_{r}^{s}\right){\bar{R}}_{c+n}^{-1}\left(\overset{⌢}{\gamma }\right)b\left(f_{t}^{s},f_{d}^{s},f_{r}^{s}\right)}.$$

where ${\bar{R}}_{c+n}^{-1}$ is expressed as ${\bar{R}}_{c+n}^{-1}\left(\overset{⌢}{\gamma }\right)$ for better understanding, because ${\bar{R}}_{c+n}^{-1}\left(\overset{⌢}{\gamma }\right)$ means that ${\bar{R}}_{c+n}^{-1}$ is a function related to the adjusted eigenvalues $\overset{⌢}{\gamma }$

Correspondingly, with the new MIMO-STAP weight vector $\bar{w}$, the radar output power is written as

(19) $$\begin{array}{ll} \rho \left(\bar{w}\left(\overset{⌢}{\gamma }\right)\right) & =\frac{b^{H}\left(f_{t}^{s},f_{d}^{s},f_{r}^{s}\right){\bar{R}}_{c+n}^{-1}\left(\overset{⌢}{\gamma }\right)R_{c+n}{\bar{R}}_{c+n}^{-1}\left(\overset{⌢}{\gamma }\right)b\left(f_{t}^{s},f_{d}^{s},f_{r}^{s}\right)}{{\left[b^{H}\left(f_{t}^{s},f_{d}^{s},f_{r}^{s}\right){\bar{R}}_{c+n}^{-1}\left(\overset{⌢}{\gamma }\right)b\left(f_{t}^{s},f_{d}^{s},f_{r}^{s}\right)\right]}^{2}}\\ & =\frac{b^{H}\left(f_{t}^{s},f_{d}^{s},f_{r}^{s}\right)\left(\sum_{l=1}^{L}{\overset{⌢}{\gamma }}_{l}d_{l}d_{l}^{H}+I_{EMK}\right)/\varepsilon_{n}\left(\sum_{l=1}^{L}{\overset{⌢}{\alpha }}_{l}u_{l}u_{l}^{H}+I_{EMK}\right)\varepsilon_{n}\left(\sum_{l=1}^{L}{\overset{⌢}{\gamma }}_{l}d_{l}d_{l}^{H}+I_{EMK}\right)/\varepsilon_{n}b\left(f_{t}^{s},f_{d}^{s},f_{r}^{s}\right)}{{\left[b^{H}\left(f_{t}^{s},f_{d}^{s},f_{r}^{s}\right)\left(\sum_{l=1}^{L}{\overset{⌢}{\gamma }}_{l}d_{l}d_{l}^{H}+I_{EMK}\right)/\varepsilon_{n}b\left(f_{t}^{s},f_{d}^{s},f_{r}^{s}\right)\right]}^{2}}\\ & =\frac{\varepsilon_{n}b^{H}\left(f_{t}^{s},f_{d}^{s},f_{r}^{s}\right)\left(\sum_{l=1}^{L}{\overset{⌢}{\gamma }}_{l}d_{l}d_{l}^{H}+I_{EMK}\right)\left(\sum_{l=1}^{L}{\overset{⌢}{\alpha }}_{l}u_{l}u_{l}^{H}+I_{EMK}\right)\left(\sum_{l=1}^{L}{\overset{⌢}{\gamma }}_{l}d_{l}d_{l}^{H}+I_{EMK}\right)b\left(f_{t}^{s},f_{d}^{s},f_{r}^{s}\right)}{{\left[b^{H}\left(f_{t}^{s},f_{d}^{s},f_{r}^{s}\right)\left(\sum_{l=1}^{L}{\overset{⌢}{\gamma }}_{l}d_{l}d_{l}^{H}+I_{EMK}\right)b\left(f_{t}^{s},f_{d}^{s},f_{r}^{s}\right)\right]}^{2}} \end{array}.$$

Then, (19) is further expressed as

(20) $$\rho \left(\bar{w}\left(\overset{⌢}{\gamma }\right)\right)=\frac{\varepsilon_{n}b^{H}\left(f_{t}^{s},f_{d}^{s},f_{r}^{s}\right)\left(\begin{array}{l} I_{EMK}+\sum_{l=1}^{L}{\overset{⌢}{\alpha }}_{l}u_{l}u_{l}^{H}+2\sum_{l=1}^{L}{\overset{⌢}{\gamma }}_{l}d_{l}d_{l}^{H}\sum_{l=1}^{L}{\overset{⌢}{\alpha }}_{l}u_{l}u_{l}^{H}\\ +2\sum_{l=1}^{L}{\overset{⌢}{\gamma }}_{l}d_{l}d_{l}^{H}+\sum_{l=1}^{L}{\overset{⌢}{\gamma }}_{l}d_{l}d_{l}^{H}\sum_{l=1}^{L}{\overset{⌢}{\gamma }}_{l}d_{l}d_{l}^{H}+\sum_{l=1}^{L}{\overset{⌢}{\gamma }}_{l}d_{l}d_{l}^{H}\sum_{l=1}^{L}{\overset{⌢}{\alpha }}_{l}u_{l}u_{l}^{H}\sum_{l=1}^{L}{\overset{⌢}{\gamma }}_{l}d_{l}d_{l}^{H} \end{array}\right)b\left(f_{t}^{s},f_{d}^{s},f_{r}^{s}\right)}{{\left[b^{H}\left(f_{t}^{s},f_{d}^{s},f_{r}^{s}\right)b\left(f_{t}^{s},f_{d}^{s},f_{r}^{s}\right)+b^{H}\left(f_{t}^{s},f_{d}^{s},f_{r}^{s}\right)\sum_{l=1}^{L}{\overset{⌢}{\gamma }}_{l}d_{l}d_{l}^{H}b\left(f_{t}^{s},f_{d}^{s},f_{r}^{s}\right)\right]}^{2}}.$$

Therefore, to design optimal EA-MIMO-STAP is to design the optimal vector ${\overset{⌢}{\gamma }}^{*}$ to minimize $\rho \left(\bar{w}\left(\overset{⌢}{\gamma }\right)\right)$, which could be expressed as

(21) $${\overset{⌢}{\gamma }}^{*}=\underset{\overset{⌢}{\gamma }}{\mathrm{argmin}}\;\rho \left(\bar{w}\left(\overset{⌢}{\gamma }\right)\right),$$

### 3.2. Asymptotic Deterministic Equivalent $\bar{\rho }\left(\bar{w}\left(\overset{⌢}{\gamma }\right)\right)$

For (21), we can see that it is considerably hard to directly derive its solution. As demonstrated in random matrix theory, if EMK,D→∞, i.e., in the asymptotic area, such an issue can be transferred to a simple form. However, in practice EMK and D will not be infinite, and this will unavoidably lead to estimation errors. Fortunately, multiple works have displayed that estimation errors are commonly acceptable [43,44,45,46]. This means that we could resort to the outcomes in the asymptotic area to improve the practical MIMO-STAP issue.

In the asymptotic area, according to the study in [48], we can have the following:

(22) $$\begin{array}{l} \left|u_{m}^{H}d_{n}d_{n}^{H}u_{m}-\varsigma_{m}\delta_{mn}\right|\overset{a.s.}{\to }0\;\;\;\;\;m,n=1,\ldots ,L\\ \left|b^{H}\left(f_{t}^{s},f_{d}^{s},f_{r}^{s}\right)d_{m}d_{m}^{H}b\left(f_{t}^{s},f_{d}^{s},f_{r}^{s}\right)-\varsigma_{m}h_{m}\right|\overset{a.s.}{\to }0\\ \left|b^{H}\left(f_{t}^{s},f_{d}^{s},f_{r}^{s}\right)d_{m}d_{m}^{H}u_{m}u_{m}^{H}b\left(f_{t}^{s},f_{d}^{s},f_{r}^{s}\right)-\varsigma_{m}h_{m}\right|\overset{a.s.}{\to }0\\ \left|b^{H}\left(f_{t}^{s},f_{d}^{s},f_{r}^{s}\right)d_{m}d_{m}^{H}u_{m}u_{m}^{H}d_{m}d_{m}^{H}b\left(f_{t}^{s},f_{d}^{s},f_{r}^{s}\right)-\varsigma_{m}^{2}h_{m}\right|\overset{a.s.}{\to }0 \end{array},$$

where $\delta_{mn}$ represents the Kronecker delta function, $\varsigma_{m}=\left(1-\frac{\zeta_{D}}{{\overset{⌢}{\alpha }}_{l}^{2}}\right)/\left(1+\frac{\zeta_{D}}{{\overset{⌢}{\alpha }}_{l}}\right)$, $\zeta_{D}=EMK/D$ and $h_{m}=b^{H}\left(f_{t}^{s},f_{d}^{s},f_{r}^{s}\right)u_{m}u_{m}^{H}b\left(f_{t}^{s},f_{d}^{s},f_{r}^{s}\right)$.

With (22), the asymptotic deterministic equivalent $\bar{\rho }\left(\bar{w}\left(\overset{⌢}{\gamma }\right)\right)$ of (20) can be mathematically expressed as

(23) $$\begin{array}{ll} \bar{\rho }\left(\bar{w}\left(\overset{⌢}{\gamma }\right)\right) & =\frac{\varepsilon_{n}\left(b^{H}\left(f_{t}^{s},f_{d}^{s},f_{r}^{s}\right)b\left(f_{t}^{s},f_{d}^{s},f_{r}^{s}\right)+2\sum_{l=1}^{L}{\overset{⌢}{\gamma }}_{l}\varsigma_{l}h_{l}+\sum_{l=1}^{L}{\overset{⌢}{\alpha }}_{l}h_{l}+2\sum_{l=1}^{L}{\overset{⌢}{\gamma }}_{l}{\overset{⌢}{\alpha }}_{l}\varsigma_{l}h_{l}+\sum_{l=1}^{L}{\overset{⌢}{\gamma }}_{l}^{2}\varsigma_{l}h_{l}+\sum_{l=1}^{L}{\overset{⌢}{\gamma }}_{l}^{2}{\overset{⌢}{\alpha }}_{l}\varsigma_{l}^{2}h_{l}\right)}{{\left[b^{H}\left(f_{t}^{s},f_{d}^{s},f_{r}^{s}\right)b\left(f_{t}^{s},f_{d}^{s},f_{r}^{s}\right)+\sum_{l=1}^{L}{\overset{⌢}{\gamma }}_{l}\varsigma_{l}h_{l}\right]}^{2}}\\ & =\frac{\varepsilon_{n}\left(b^{H}\left(f_{t}^{s},f_{d}^{s},f_{r}^{s}\right)b\left(f_{t}^{s},f_{d}^{s},f_{r}^{s}\right)+\sum_{l=1}^{L}\left({\overset{⌢}{\gamma }}_{l}^{2}{\overset{⌢}{\alpha }}_{l}\varsigma_{l}^{2}h_{l}+{\overset{⌢}{\gamma }}_{l}^{2}\varsigma_{l}h_{l}+2{\overset{⌢}{\gamma }}_{l}{\overset{⌢}{\alpha }}_{l}\varsigma_{l}h_{l}+2{\overset{⌢}{\gamma }}_{l}\varsigma_{l}h_{l}+{\overset{⌢}{\alpha }}_{l}h_{l}\right)\right)}{{\left[b^{H}\left(f_{t}^{s},f_{d}^{s},f_{r}^{s}\right)b\left(f_{t}^{s},f_{d}^{s},f_{r}^{s}\right)+\sum_{l=1}^{L}{\overset{⌢}{\gamma }}_{l}\varsigma_{l}h_{l}\right]}^{2}} \end{array}.$$

It should be noted that in practical applications of airborne radars, the system DoF is commonly large since the numbers of transmit antenna elements, receive antenna elements and pulses are sufficiently large. Therefore, it is reasonable to resort to the outcomes in the asymptotic area.

### 3.3. Optimized Solution ${\bar{\overset{⌢}{\gamma }}}^{*}$ Derivation

Let $\bar{\rho }\left(\bar{w}\left(\overset{⌢}{\gamma }\right)\right)$ take the place of $\rho \left(\bar{w}\left(\overset{⌢}{\gamma }\right)\right)$ in (21); the optimized solution ${\bar{\overset{⌢}{\gamma }}}^{*}={\left[{\bar{\overset{⌢}{\gamma }}}_{1}^{*},\cdots ,{\bar{\overset{⌢}{\gamma }}}_{L}^{*}\right]}^{T}$ can then be calculated.

(24) $$\begin{array}{ll} \bar{\rho }\left(\bar{w}\left(\overset{⌢}{\gamma }\right)\right) & =\varepsilon_{n}\frac{\sum_{l=1}^{L}\varsigma_{l}h_{l}\left(1+\varsigma_{l}{\overset{⌢}{\alpha }}_{l}\right){\left({\overset{⌢}{\gamma }}_{l}+\frac{1+{\overset{⌢}{\alpha }}_{l}}{1+\varsigma_{l}{\overset{⌢}{\alpha }}_{l}}\right)}^{2}+\sum_{l=1}^{L}\left[h_{l}{\overset{⌢}{\alpha }}_{l}-\frac{\varsigma_{l}h_{l}{\left(1+{\overset{⌢}{\alpha }}_{l}\right)}^{2}}{1+\varsigma_{l}{\overset{⌢}{\alpha }}_{l}}\right]}{{\left[\sum_{e=1}^{G}\varsigma_{l}h_{l}\left({\overset{⌢}{\gamma }}_{l}+\frac{1+{\overset{⌢}{\alpha }}_{l}}{1+\varsigma_{l}{\overset{⌢}{\alpha }}_{l}}\right)+b^{H}\left(f_{t}^{s},f_{d}^{s},f_{r}^{s}\right)b\left(f_{t}^{s},f_{d}^{s},f_{r}^{s}\right)-\frac{\varsigma_{l}h_{l}\left(1+{\overset{⌢}{\alpha }}_{l}\right)}{1+\varsigma_{l}{\overset{⌢}{\alpha }}_{l}}\right]}^{2}}\\ & =\varepsilon_{n}\frac{\sum_{l=1}^{L}\varsigma_{l}h_{l}\left(1+\varsigma_{l}{\overset{⌢}{\alpha }}_{l}\right){\left({\overset{⌢}{\gamma }}_{l}+\frac{1+{\overset{⌢}{\alpha }}_{l}}{1+\varsigma_{l}{\overset{⌢}{\alpha }}_{l}}\right)}^{2}+\sum_{l=1}^{L}\left[h_{l}{\overset{⌢}{\alpha }}_{l}-\frac{\varsigma_{l}h_{l}{\left(1+{\overset{⌢}{\alpha }}_{l}\right)}^{2}}{1+\varsigma_{l}{\overset{⌢}{\alpha }}_{l}}\right]}{{\left[\begin{array}{l} \sum_{e=1}^{G}\sqrt{\varsigma_{l}h_{l}\left(1+\varsigma_{l}{\overset{⌢}{\alpha }}_{l}\right)}\left({\overset{⌢}{\gamma }}_{l}+\frac{1+{\overset{⌢}{\alpha }}_{l}}{1+\varsigma_{l}{\overset{⌢}{\alpha }}_{l}}\right)\frac{\varsigma_{l}h_{l}}{\sqrt{\varsigma_{l}h_{l}\left(1+\varsigma_{l}{\overset{⌢}{\alpha }}_{l}\right)}}+\\ \sqrt{\sum_{l=1}^{L}\left[h_{l}{\overset{⌢}{\alpha }}_{l}-\frac{\varsigma_{l}h_{l}{\left(1+{\overset{⌢}{\alpha }}_{l}\right)}^{2}}{1+\varsigma_{l}{\overset{⌢}{\alpha }}_{l}}\right]}\frac{b^{H}\left(f_{t}^{s},f_{d}^{s},f_{r}^{s}\right)b\left(f_{t}^{s},f_{d}^{s},f_{r}^{s}\right)-\frac{\varsigma_{l}h_{l}\left(1+{\overset{⌢}{\alpha }}_{l}\right)}{1+\varsigma_{l}{\overset{⌢}{\alpha }}_{l}}}{\sqrt{\sum_{l=1}^{L}\left[h_{l}{\overset{⌢}{\alpha }}_{l}-\frac{\varsigma_{l}h_{l}{\left(1+{\overset{⌢}{\alpha }}_{l}\right)}^{2}}{1+\varsigma_{l}{\overset{⌢}{\alpha }}_{l}}\right]}} \end{array}\right]}^{2}} \end{array}.$$

By re-writing (24), we will have the minimal value of $\bar{\rho }\left(\bar{w}\left(\overset{⌢}{\gamma }\right)\right)$ in (23):

(25) $$\begin{array}{l} \bar{\rho }\left(\bar{w}\left(\overset{⌢}{\gamma }\right)\right)=\varepsilon_{n}\frac{\sum_{l=1}^{L}\varsigma_{l}h_{l}\left(1+\varsigma_{l}{\overset{⌢}{\alpha }}_{l}\right){\left({\overset{⌢}{\gamma }}_{l}+\frac{1+{\overset{⌢}{\alpha }}_{l}}{1+\varsigma_{l}{\overset{⌢}{\alpha }}_{l}}\right)}^{2}+\sum_{l=1}^{L}\left[h_{l}{\overset{⌢}{\alpha }}_{l}-\frac{\varsigma_{l}h_{l}{\left(1+{\overset{⌢}{\alpha }}_{l}\right)}^{2}}{1+\varsigma_{l}{\overset{⌢}{\alpha }}_{l}}\right]+b^{H}\left(f_{t}^{s},f_{d}^{s},f_{r}^{s}\right)b\left(f_{t}^{s},f_{d}^{s},f_{r}^{s}\right)}{{\left[\begin{array}{l} \sum_{e=1}^{G}\sqrt{\varsigma_{l}h_{l}\left(1+\varsigma_{l}{\overset{⌢}{\alpha }}_{l}\right)}\left({\overset{⌢}{\gamma }}_{l}+\frac{1+{\overset{⌢}{\alpha }}_{l}}{1+\varsigma_{l}{\overset{⌢}{\alpha }}_{l}}\right)\frac{\varsigma_{l}h_{l}}{\sqrt{\varsigma_{l}h_{l}\left(1+\varsigma_{l}{\overset{⌢}{\alpha }}_{l}\right)}}+\\ \sqrt{\sum_{l=1}^{L}\begin{array}{l} \left[h_{l}{\overset{⌢}{\alpha }}_{l}-\frac{\varsigma_{l}h_{l}{\left(1+{\overset{⌢}{\alpha }}_{l}\right)}^{2}}{1+\varsigma_{l}{\overset{⌢}{\alpha }}_{l}}\right]+\\ b^{H}\left(f_{t}^{s},f_{d}^{s},f_{r}^{s}\right)b\left(f_{t}^{s},f_{d}^{s},f_{r}^{s}\right) \end{array}}\frac{b^{H}\left(f_{t}^{s},f_{d}^{s},f_{r}^{s}\right)b\left(f_{t}^{s},f_{d}^{s},f_{r}^{s}\right)-\frac{\varsigma_{l}h_{l}\left(1+{\overset{⌢}{\alpha }}_{l}\right)}{1+\varsigma_{l}{\overset{⌢}{\alpha }}_{l}}}{\sqrt{\begin{array}{l} \sum_{l=1}^{L}\left[h_{l}{\overset{⌢}{\alpha }}_{l}-\frac{\varsigma_{l}h_{l}{\left(1+{\overset{⌢}{\alpha }}_{l}\right)}^{2}}{1+\varsigma_{l}{\overset{⌢}{\alpha }}_{l}}\right]+\\ b^{H}\left(f_{t}^{s},f_{d}^{s},f_{r}^{s}\right)b\left(f_{t}^{s},f_{d}^{s},f_{r}^{s}\right) \end{array}}} \end{array}\right]}^{2}}\\ \geq \varepsilon_{n}\frac{\sum_{l=1}^{L}\varsigma_{l}h_{l}\left(1+\varsigma_{l}{\overset{⌢}{\alpha }}_{l}\right){\left({\overset{⌢}{\gamma }}_{l}+\frac{1+{\overset{⌢}{\alpha }}_{l}}{1+\varsigma_{l}{\overset{⌢}{\alpha }}_{l}}\right)}^{2}+\sum_{l=1}^{L}\left[h_{l}{\overset{⌢}{\alpha }}_{l}-\frac{\varsigma_{l}h_{l}{\left(1+{\overset{⌢}{\alpha }}_{l}\right)}^{2}}{1+\varsigma_{l}{\overset{⌢}{\alpha }}_{l}}\right]+b^{H}\left(f_{t}^{s},f_{d}^{s},f_{r}^{s}\right)b\left(f_{t}^{s},f_{d}^{s},f_{r}^{s}\right)}{\left[\begin{array}{l} \sum_{l=1}^{L}\varsigma_{l}h_{l}\left(1+\varsigma_{l}{\overset{⌢}{\alpha }}_{l}\right){\left({\overset{⌢}{\gamma }}_{l}+\frac{1+{\overset{⌢}{\alpha }}_{l}}{1+\varsigma_{l}{\overset{⌢}{\alpha }}_{l}}\right)}^{2}\\ +\sum_{l=1}^{L}\left[h_{l}{\overset{⌢}{\alpha }}_{l}-\frac{\varsigma_{l}h_{l}{\left(1+{\overset{⌢}{\alpha }}_{l}\right)}^{2}}{1+\varsigma_{l}{\overset{⌢}{\alpha }}_{l}}\right] \end{array}\right]\left[\sum_{l=1}^{L}\frac{{\left(\varsigma_{l}h_{l}\right)}^{2}}{\varsigma_{l}h_{l}\left(1+\varsigma_{l}{\overset{⌢}{\alpha }}_{l}\right)}+\frac{{\left(\begin{array}{l} b^{H}\left(f_{t}^{s},f_{d}^{s},f_{r}^{s}\right)b\left(f_{t}^{s},f_{d}^{s},f_{r}^{s}\right)-\\ \frac{\varsigma_{l}h_{l}\left(1+{\overset{⌢}{\alpha }}_{l}\right)}{1+\varsigma_{l}{\overset{⌢}{\alpha }}_{l}} \end{array}\right)}^{2}}{\begin{array}{l} \sum_{l=1}^{L}\left[h_{l}{\overset{⌢}{\alpha }}_{l}-\frac{\varsigma_{l}h_{l}{\left(1+{\overset{⌢}{\alpha }}_{l}\right)}^{2}}{1+\varsigma_{l}{\overset{⌢}{\alpha }}_{l}}\right]+\\ b^{H}\left(f_{t}^{s},f_{d}^{s},f_{r}^{s}\right)b\left(f_{t}^{s},f_{d}^{s},f_{r}^{s}\right) \end{array}}\right]}\\ =\frac{\varepsilon_{n}}{\sum_{l=1}^{L}\frac{{\left(\varsigma_{l}h_{l}\right)}^{2}}{\varsigma_{l}h_{l}\left(1+\varsigma_{l}{\overset{⌢}{\alpha }}_{l}\right)}+\frac{{\left(\begin{array}{l} b^{H}\left(f_{t}^{s},f_{d}^{s},f_{r}^{s}\right)b\left(f_{t}^{s},f_{d}^{s},f_{r}^{s}\right)-\\ \frac{\varsigma_{l}h_{l}\left(1+{\overset{⌢}{\alpha }}_{l}\right)}{1+\varsigma_{l}{\overset{⌢}{\alpha }}_{l}} \end{array}\right)}^{2}}{\sum_{l=1}^{L}\left[h_{l}{\overset{⌢}{\alpha }}_{l}-\frac{\varsigma_{l}h_{l}{\left(1+{\overset{⌢}{\alpha }}_{l}\right)}^{2}}{1+\varsigma_{l}{\overset{⌢}{\alpha }}_{l}}\right]+b^{H}\left(f_{t}^{s},f_{d}^{s},f_{r}^{s}\right)b\left(f_{t}^{s},f_{d}^{s},f_{r}^{s}\right)}} \end{array}.$$

In (25), we find that only the following equality is satisfied, and the minimal value can be achieved:

(26) $$\frac{\sqrt{\varsigma_{l}h_{l}\left(1+\varsigma_{l}{\overset{⌢}{\alpha }}_{l}\right)}\left({\overset{⌢}{\gamma }}_{l}+\frac{1+{\overset{⌢}{\alpha }}_{l}}{1+\varsigma_{l}{\overset{⌢}{\alpha }}_{l}}\right)\left(b^{H}\left(f_{t}^{s},f_{d}^{s},f_{r}^{s}\right)b\left(f_{t}^{s},f_{d}^{s},f_{r}^{s}\right)-\frac{\varsigma_{l}h_{l}\left(1+{\overset{⌢}{\alpha }}_{l}\right)}{1+\varsigma_{l}{\overset{⌢}{\alpha }}_{l}}\right)}{\sqrt{\sum_{l=1}^{L}\left[h_{l}{\overset{⌢}{\alpha }}_{l}-\frac{\varsigma_{l}h_{l}{\left(1+{\overset{⌢}{\alpha }}_{l}\right)}^{2}}{1+\varsigma_{l}{\overset{⌢}{\alpha }}_{l}}\right]+b^{H}\left(f_{t}^{s},f_{d}^{s},f_{r}^{s}\right)b\left(f_{t}^{s},f_{d}^{s},f_{r}^{s}\right)}}=\frac{\varsigma_{l}h_{l}\sqrt{\begin{array}{l} \sum_{l=1}^{L}\left[h_{l}{\overset{⌢}{\alpha }}_{l}-\frac{\varsigma_{l}h_{l}{\left(1+{\overset{⌢}{\alpha }}_{l}\right)}^{2}}{1+\varsigma_{l}{\overset{⌢}{\alpha }}_{l}}\right]+\\ b^{H}\left(f_{t}^{s},f_{d}^{s},f_{r}^{s}\right)b\left(f_{t}^{s},f_{d}^{s},f_{r}^{s}\right) \end{array}}}{\sqrt{\varsigma_{l}h_{l}\left(1+\varsigma_{l}{\overset{⌢}{\alpha }}_{l}\right)}}.$$

Therefore, with (26), the optimized solution ${\bar{\overset{⌢}{\gamma }}}_{l}^{*}$ can be calculated as

(27) $$\begin{array}{cc} {\bar{\overset{⌢}{\gamma }}}_{l}^{*} & =\frac{\varsigma_{l}h_{l}\left(\sum_{l=1}^{L}\left[h_{l}{\overset{⌢}{\alpha }}_{l}-\frac{\varsigma_{l}h_{l}{\left(1+{\overset{⌢}{\alpha }}_{l}\right)}^{2}}{1+\varsigma_{l}{\overset{⌢}{\alpha }}_{l}}\right]+b^{H}\left(f_{t}^{s},f_{d}^{s},f_{r}^{s}\right)b\left(f_{t}^{s},f_{d}^{s},f_{r}^{s}\right)\right)}{\varsigma_{l}h_{l}\left(1+\varsigma_{l}{\overset{⌢}{\alpha }}_{l}\right)\left(b^{H}\left(f_{t}^{s},f_{d}^{s},f_{r}^{s}\right)b\left(f_{t}^{s},f_{d}^{s},f_{r}^{s}\right)-\frac{\varsigma_{l}h_{l}\left(1+{\overset{⌢}{\alpha }}_{l}\right)}{1+\varsigma_{l}{\overset{⌢}{\alpha }}_{l}}\right)}-\frac{1+{\overset{⌢}{\alpha }}_{l}}{1+\varsigma_{l}{\overset{⌢}{\alpha }}_{l}}\\ & =\frac{{\overset{⌢}{\alpha }}_{l}+\zeta_{D}}{{\overset{⌢}{\alpha }}_{l}^{2}+{\overset{⌢}{\alpha }}_{l}}\left[\frac{\sum_{i=1}^{L}\frac{\zeta_{D}h_{i}}{{\overset{⌢}{\alpha }}_{i}}}{b^{H}\left(f_{t}^{s},f_{d}^{s},f_{r}^{s}\right)b\left(f_{t}^{s},f_{d}^{s},f_{r}^{s}\right)-\sum_{i=1}^{L}h_{i}+\sum_{i=1}^{L}\frac{\zeta_{D}h_{i}}{{\overset{⌢}{\alpha }}_{i}^{2}}}-{\overset{⌢}{\alpha }}_{l}\right] \end{array}.$$

### 3.4. Estimation $\bar{w}\left(\hat{\overset{⌢}{\gamma }}\right)$ of MIMO-STAP Weight

Although (27) was derived for optimized MIMO-STAP, we can see that ${\bar{\overset{⌢}{\gamma }}}_{l}^{*}$ requires ${\overset{⌢}{\alpha }}_{l}$ and $h_{l}$ first, and this is impossible in practical scenarios because they involve $\alpha_{l}$ and $u_{l}$. Therefore, we need to calculate consistent estimations of ${\overset{⌢}{\alpha }}_{e}$ and $h_{l}$. In terms of the outcomes on isolated eigenvalues of the spiked framework [43,44,45,46], there is a one-to-one mapping between sample eigenvalues ${\overset{⌢}{\beta }}_{l}$ and real eigenvalues ${\overset{⌢}{\alpha }}_{l}$ $\left(l=1,\ldots ,L\right)$. Nonetheless, the implementation of the mapping needs to satisfy an assumption:

**A.**  ${\overset{⌢}{\alpha }}_{1}\geq \cdots \geq {\overset{⌢}{\alpha }}_{L}>\sqrt{\zeta }$ and ${\overset{⌢}{\beta }}_{1}\geq \cdots \geq {\overset{⌢}{\beta }}_{L}>{\left(1+\sqrt{\zeta }\right)}^{2}$

**Remark** **1.** *Assumption A represents the condition of recovery of isolated eigenvalues in the spike model. On the one hand, ${\overset{⌢}{\alpha }}_{1}\geq \cdots \geq {\overset{⌢}{\alpha }}_{L}>\sqrt{\zeta }$ is in practice easy to satisfy since the clutter eigenvalues ${\overset{⌢}{\alpha }}_{l}$ corresponding to clutter power are generally larger than the noise eigenvalues corresponding to noise power. On the other hand, for ${\overset{⌢}{\beta }}_{1}\geq \cdots \geq {\overset{⌢}{\beta }}_{L}>{\left(1+\sqrt{\zeta }\right)}^{2}$, the number of snapshots D is supposed to exceed the clutter rank L. Otherwise, there will be zero eigenvalues in ${\overset{⌢}{\beta }}_{l}\left(l=D+1,\cdots ,L\right)$, hampering the isolated eigenvalue estimation.*

Then, under assumption A, according to the outcomes in [43], the mapping between ${\overset{⌢}{\alpha }}_{l}$ and ${\overset{⌢}{\beta }}_{l}$ is described as

(28) $$\begin{array}{l} \left|{\overset{⌢}{\beta }}_{l}-{\overset{⌢}{\alpha }}_{l}-\frac{\zeta_{D}\left(1+{\overset{⌢}{\alpha }}_{l}\right)}{{\overset{⌢}{\alpha }}_{l}}-1\right|\overset{a.s.}{\to }0\\ \left|h_{l}-\frac{1+\frac{\zeta_{D}}{{\hat{\overset{⌢}{\alpha }}}_{l}}}{1-\frac{\zeta_{D}}{{\hat{\overset{⌢}{\alpha }}}_{l}^{2}}}b^{H}\left(f_{t}^{s},f_{d}^{s},f_{r}^{s}\right)d_{l}d_{l}^{H}b\left(f_{t}^{s},f_{d}^{s},f_{r}^{s}\right)\right|\overset{a.s.}{\to }0 \end{array}.$$

Then, with the outcomes in (28), consistent estimations of ${\overset{⌢}{\alpha }}_{l}$ and $h_{l}$ are calculated as

(29) $$\begin{array}{l} {\hat{\overset{⌢}{\alpha }}}_{l}=\frac{{\overset{⌢}{\beta }}_{l}-1+\sqrt{{\left({\overset{⌢}{\beta }}_{l}-1-\zeta_{D}\right)}^{2}-4\zeta_{D}}-\zeta_{D}}{2}\\ {\hat{h}}_{l}=\frac{1+\frac{\zeta_{D}}{{\hat{\overset{⌢}{\alpha }}}_{l}}}{1-\frac{\zeta_{D}}{{\hat{\overset{⌢}{\alpha }}}_{l}^{2}}}b^{H}\left(f_{t}^{s},f_{d}^{s},f_{r}^{s}\right)d_{l}d_{l}^{H}b\left(f_{t}^{s},f_{d}^{s},f_{r}^{s}\right) \end{array}.$$

With (29), let the estimated ${\hat{\overset{⌢}{\alpha }}}_{l}$ and ${\hat{h}}_{l}$ replace ${\overset{⌢}{\alpha }}_{l}$ and $h_{l}$ in (27); we can then estimate the optimal ${\hat{\overset{⌢}{\gamma }}}_{l}^{*}$ as

(30) $${\hat{\overset{⌢}{\gamma }}}_{l}^{*}=\frac{{\hat{\overset{⌢}{\alpha }}}_{l}+\zeta_{D}}{{\hat{\overset{⌢}{\alpha }}}_{l}^{2}+{\hat{\overset{⌢}{\alpha }}}_{l}}\left[\frac{\sum_{i=1}^{L}\frac{\zeta_{D}{\hat{h}}_{i}}{{\hat{\overset{⌢}{\alpha }}}_{i}}}{b^{H}\left(f_{t}^{s},f_{d}^{s},f_{r}^{s}\right)b\left(f_{t}^{s},f_{d}^{s},f_{r}^{s}\right)-\sum_{i=1}^{L}{\hat{h}}_{i}+\sum_{i=1}^{L}\frac{\zeta_{D}{\hat{h}}_{i}}{{\hat{\overset{⌢}{\alpha }}}_{i}^{2}}}-{\hat{\overset{⌢}{\alpha }}}_{l}\right].$$

Correspondingly, by replacing ${\bar{\overset{⌢}{\gamma }}}_{l}^{*}$ with ${\hat{\overset{⌢}{\gamma }}}_{l}^{*}$, the estimated optimal ${\bar{R}}_{c+n}^{-1}\left({\hat{\overset{⌢}{\gamma }}}^{*}\right)$ can be derived as

(31) $${\bar{R}}_{c+n}^{-1}\left({\hat{\overset{⌢}{\gamma }}}^{*}\right)=\left(\sum_{l=1}^{L}{\hat{\overset{⌢}{\gamma }}}_{l}^{*}d_{l}d_{l}^{H}+I_{EMK}\right)/\varepsilon_{n},$$

Consequently, the proposed EA-MIMO-STAP can be derived as

(32) $$\bar{w}\left({\hat{\overset{⌢}{\gamma }}}^{*}\right)=\frac{{\bar{R}}_{c+n}^{-1}\left({\hat{\overset{⌢}{\gamma }}}^{*}\right)b\left(f_{t}^{s},f_{d}^{s},f_{r}^{s}\right)}{b^{H}\left(f_{t}^{s},f_{d}^{s},f_{r}^{s}\right){\bar{R}}_{c+n}^{-1}\left({\hat{\overset{⌢}{\gamma }}}^{*}\right)b\left(f_{t}^{s},f_{d}^{s},f_{r}^{s}\right)}.$$

In (17) and (30), it is assumed that the noise power $\varepsilon_{n}$ has been effectively estimated. In practice, many measures are given to estimate this parameter [32,33,49]. In this study, we adopt an approach where the sample noise eigenvalues are averaged to obtain an estimate of $\varepsilon_{n}$ [32]:

(33) $$\varepsilon_{n}=\frac{1}{EMK-L}\sum_{l=L+1}^{EMK}\beta_{l}.$$

### 3.5. Computational Complexity

In the context of STAP, the computational complexity is mainly attributed to matrix inversion and eigen-decomposition [10]. For comparison, we present the computational loads associated with several classical MIMO-STAP approaches, i.e., optimal MIMO-STAP, FD-MIMO-STAP, RD-MIMO-STAP and EA-MIMO-STAP.

As elaborated in Section 2, both FD-MIMO-STAP and optimal MIMO-STAP are formulated based on the inversion of the CPNCM. Consequently, their computational loads scale with the cube of the system DoF, i.e., they are proportional to $O\left(E^{3}M^{3}K^{3}\right)$, where EMK represents the system DoF. Furthermore, as detailed in Section 3.1, EA-MIMO-STAP is implemented through the eigen-decomposition of the CPNCM. Similarly to that of the aforementioned methods, its computational load scales with the cube of the system DoF, i.e., $O\left(E^{3}M^{3}K^{3}\right)$. However, it is noteworthy that for EA-MIMO-STAP, an additional computational overhead arises from the calculation of ${\hat{\overset{⌢}{\gamma }}}_{l}^{*}$ in (30), $O\left(L^{2}\right)$, where L is far less than EMK. Nevertheless, in practical applications, this extra computational load introduced by EA-MIMO-STAP is relatively modest. For RD-MIMO-STAP, which employs the joint domain located (JDL) MIMO-STAP representation, assuming the selection of $\bar{E}$ transmit channels, $\bar{M}$ Doppler channels, and $\bar{K}$ receive channels, the system DoF amounts to $\bar{E}\bar{M}\bar{K}$. Consequently, the computational load associated with RD-MIMO-STAP is $O\left({\bar{E}}^{3}{\bar{M}}^{3}{\bar{K}}^{3}\right)$.

## 4. Numerical Experiments

This section presents a series of experimental evaluations aimed at demonstrating the performance advantages of EA-MIMO-STAP. Table 1 details the parameters employed in the experimental scenarios, and we verified that the proposed EA-MIMO-STAP can still work properly when these parameters vary. Specifically, classical RD-MIMO-STAP, the JDL method, was utilized with predefined values $\bar{E}=3$, $\bar{M}=3$ and $\bar{K}=3$. To show the advantages of the EA-MIMO-STAP algorithm in SINR improvement across varying clutter conditions, the platform speed V and the transmit antenna element spacing $\sigma_{T}$ were set to different values. Furthermore, both real and estimated noise powers were incorporated into the numerical experiments for rigorous verification of the robustness of the EA-MIMO-STAP algorithm.

Here, the output SINR loss serves as a key metric for evaluating different MIMO-STAP methods [10]. To illustrate, the output SINR loss for the FD-MIMO-STAP method is expressed as follows:

(34) $$SINR_{Loss}^{FD-MIMO-STAP}=\frac{\varepsilon_{n}}{EMK}\frac{{\left|{\hat{w}}^{H}b\left(f_{t}^{s},f_{d}^{s},f_{r}^{s}\right)\right|}^{2}}{{\hat{w}}^{H}R_{c+n}\hat{w}}.$$

Comparable output SINR losses were observed for the RD-MIMO-STAP, EA-MIMO-STAP, and optimal MIMO-STAP algorithms. To ensure statistical robustness, the output SINR losses were computed via averaging 200 Monte Carlo simulations.

**Figure 3:**
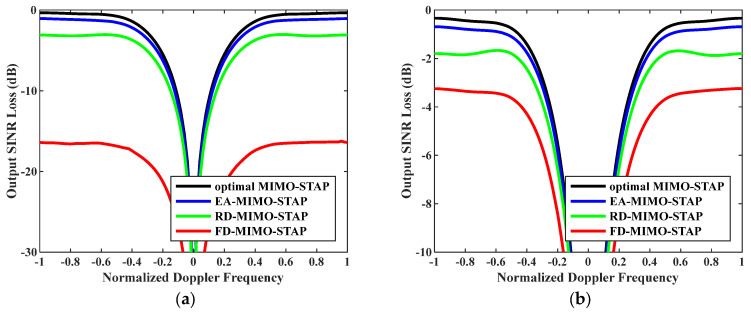
Output SINR losses versus normalized Doppler frequencies with V=150 m/s, $\sigma_{T}=0.144\;m$ and real noise power: (**a**) D=EMK and (**b**) D=2EMK.

Firstly, the relationship between output SINR losses and normalized Doppler frequencies is presented in Figure 3. The amounts of snapshots were set to D=EMK and D=2EMK. To accurately assess EA-MIMO-STAP’s performance, real noise power was utilized in the evaluation. The platform speed was configured to V=150 m/s as indicated, and the transmit antenna element spacing was set to $\sigma_{T}=0.144\;m$. The proposed EA-MIMO-STAP algorithm showed superior performance compared to the conventional FD-MIMO-STAP and RD-MIMO-STAP approaches, particularly for limited snapshots. In Figure 3a, where D=EMK, the proposed EA-MIMO-STAP algorithm exhibits substantial performance benefits, surpassing conventional FD-MIMO-STAP. Specifically, it achieved a notable reduction in output SINR loss, with declines of about 17 dB and 3 dB compared to FD-MIMO-STAP and RD-MIMO-STAP, respectively. In Figure 3b, where D=2EMK, the number of snapshots adheres to the RMB rule. The proposed algorithm demonstrates a reduced output SINR loss relative to that in Figure 3a. Despite the notable performance improvements observed in the conventional FD-MIMO-STAP approach, the proposed EA-MIMO-STAP method still outperformed it. Specifically, when compared to both RD-MIMO-STAP and conventional FD-MIMO-STAP, EA-MIMO-STAP exhibited the highest performance, with its SINR loss showing reductions of approximately 1.6 dB relative to RD-MIMO-STAP and 2.8 dB relative to FD-MIMO-STAP.

**Figure 4:**
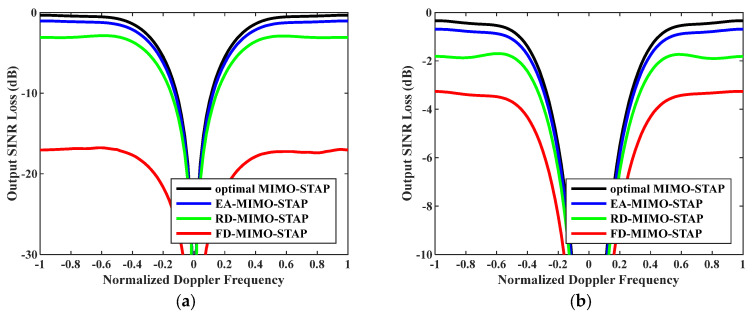
Output SINR losses versus normalized Doppler frequencies with V=150 m/s, $\sigma_{T}=0.288\;m$ and estimated noise power: (**a**) D=EMK and (**b**) D=2EMK.

Secondly, the relationship between output SINR losses and normalized Doppler frequencies is presented in Figure 4, where the numbers of snapshots were set to D=EMK and D=2EMK. As is known, we practically have no access to the precise noise power; therefore, we exploited Equation (33) to calculate the noise power to reveal the robustness of the EA-MIMO-STAP algorithm. The platform speed was configured to V=150 m/s as indicated, and the transmit antenna element spacing was set to $\sigma_{T}=0.144\;m$. It is evident that the proposed EA-MIMO-STAP had comparable performance to that depicted in Figure 3, with only minimal performance degradation. Moreover, regardless of whether the amount of snapshots conformed to the RMB rule, the EA-MIMO-STAP algorithm consistently exhibited the lowest output SINR loss when compared to both RD-MIMO-STAP and conventional FD-MIMO-STAP, thereby achieving near-optimal performance.

**Figure 5:**
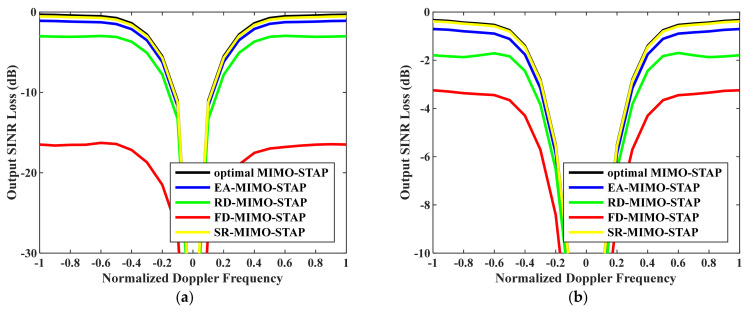
Output SINR losses versus normalized Doppler frequencies: (**a**) D=EMK; (**b**) D=2EMK.

Thirdly, to demonstrate the effectiveness of our proposed EA-MIMO-STAP, we also compare it with classical SR-MIMO-STAP in Figure 5. As an effective SR method, atomic norm minimization receives much attention due to its superior performance. Therefore, atomic norm minimization was considered as the representation of SR-MIMO-STAP. In the experiment, V=150 m/s and $\sigma_{T}=0.144\;m$ were set. The numbers of snapshots were set to D=EMK and D=2EMK. It can be seen that SR-MIMO-STAP had the best performance due to its accurate estimation of the CPNCM with its superior sparse recovery algorithm, the performance of which was close to that of the optimal MIMO-STAP. On the other hand, the proposed EA-MIMO-STAP also had great performance though it had a small performance decline compared to SR-MIMO-STAP. We can see that EA-MIMO-STAP has distinct performance advantages in comparison with FD-MIMO-STAP and RD-MIMO-STAP.

**Figure 6:**
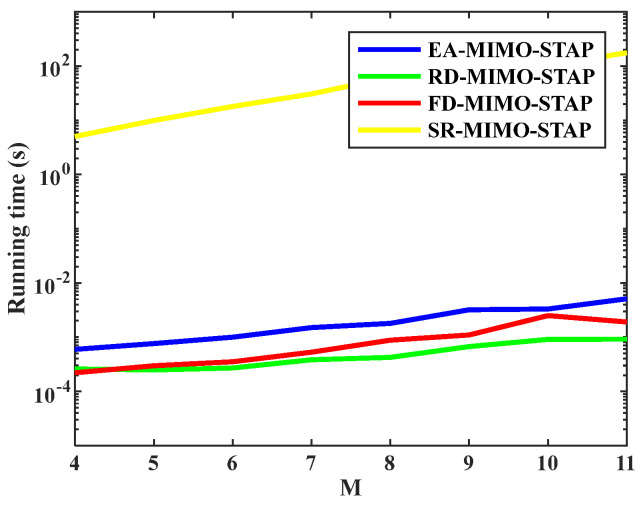
Running time versus number of pulses.

Fourthly, in STAP, it is well-known that computational time commonly decides the availability of STAP algorithms in multiple cases. To demonstrate the superiority of the proposed EA-MIMO-STAP in terms of computational efficiency, we compared the computational times of EA-MIMO-STAP, RD-MIMO-STAP, FD-MIMO-STAP, and SR-MIMO-STAP to evaluate their efficiency. The computational platform was an i7-8550U processor and MATLAB 2021a. The relationship between the running time and pulse number is shown in Figure 6, and the other parameters were set the same as those for Figure 5. It was found that, for RD-MIMO-STAP, the running time was lowest because it had the minimal system DoF due to the decrease in dimension; FD-MIMO-STAP and the proposed EA-MIMO-STAP had similar running times. SR-MIMO-STAP had the highest running time, and this running time could not be accepted in a real-time environment. Currently, SR-MIMO-STAP is commonly not applied in airborne radar. Therefore, in the following experiments, we only compared EA-MIMO-STAP, RD-MIMO-STAP and FD-MIMO-STAP.

**Figure 7:**
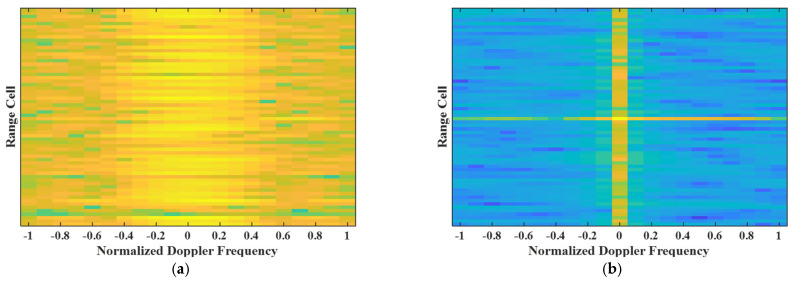
Range–Doppler spectra before and after clutter rejection: (**a**) before clutter rejection; (**b**) after clutter rejection.

Fifthly, the range–Doppler spectrum is also a metric for evaluating MIMO-STAP performance. To further illustrate the advantages of the proposed EA-MIMO-STAP method, in Figure 7 we present the range–Doppler spectra before and after clutter rejection for the proposed EA-MIMO-STAP algorithm. In this experiment, the parameters were the same as those in the second experiment. It was found that before clutter rejection, the clutter was distributed in a wide scope of the temporal domain in each range cell, while after processing by the proposed EA-MIMO-STAP algorithm, the clutter was effectively rejected.

**Figure 8:**
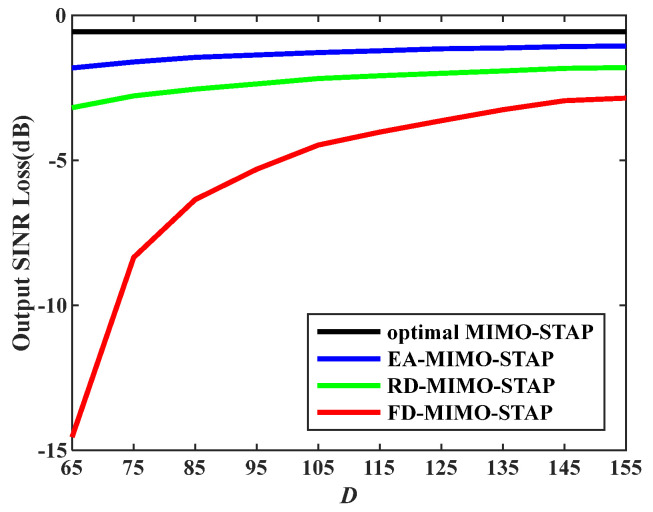
Output SINR losses versus D.

Sixthly, Figure 8 illustrates the output SINR losses plotted against the amount of snapshots D with normalized Doppler frequency $f_{d}^{s}=0.8$, V=150 m/s, and $\sigma_{T}=0.144\;m$. It can be observed that when the number D is large, EA-MIMO-STAP, RD-MIMO-STAP, and conventional FD-MIMO-STAP exhibit equivalent performance, as the RMB rule is fulfilled. However, as the number D declines and the RMB rule cannot be satisfied, the proposed EA-MIMO-STAP algorithm demonstrates distinct performance advantages over both RD-MIMO-STAP and the conventional FD-MIMO-STAP. Together with the insights from Figure 4, this experiment further corroborates the superior performance of EA-MIMO-STAP, particularly under conditions of limited snapshots.

**Figure 9:**
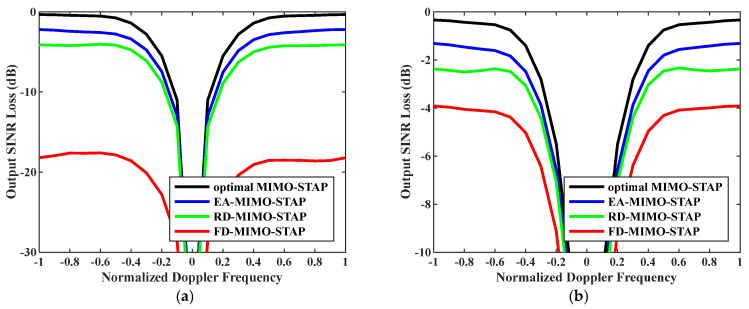
Output SINR losses versus normalized Doppler frequencies: (**a**) D=EMK; (**b**) D=2EMK.

Seventhly, to verify the effectiveness of the proposed EA-MIMO-STAP in heterogeneous environments, we simulated the hybrid clutter model with mountains and irregular vegetation according to [50]. The output SINR loss versus normalized Doppler frequencies is presented in Figure 9. In the experiments, the numbers of snapshots were set to D=EMK and D=2EMK. V=150 m/s and $\sigma_{T}=0.144\;m$ were set. Compared with Figure 4, this figure showed that MIMO-STAP had a small performance decrease. Nevertheless, it was found that the proposed EA-MIMO-STAP still performed better than the other methods.

**Figure 10:**
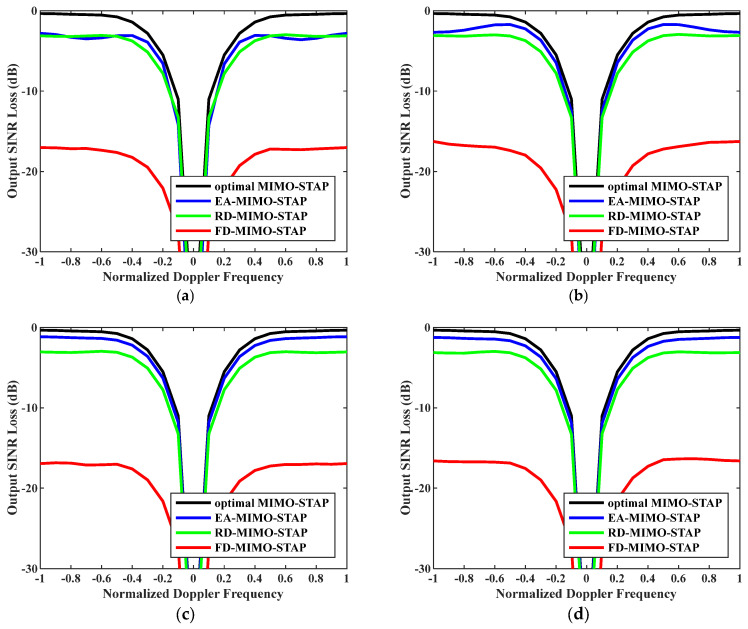
Output SINR losses versus normalized Doppler frequencies: (**a**) *L* = 8; (**b**) *L* = 9; (**c**) *L* = 11 and (**d**) *L* = 12.

Eighthly, to demonstrate the robustness of the proposed EA-MIMO-STAP to inaccurate clutter rank estimation, we considered the situation of clutter rank mismatch. In the experiment, V=150 m/s and $\sigma_{T}=0.144\;m$ were set. The numbers of snapshots were set to D=EMK. In this case, the clutter rank could be calculated as 10. We give two simulation results with *L* = 8, *L* = 9, *L* = 11 and *L* = 12 in Figure 10. It can be seen that the proposed EA-MIMO-STAP maintained superior performance. When *L* = 8, the performance of the proposed EA-MIMO-STAP was similar to that of RD-MIMO-STAP, but when *L* was less than 8, EA-MIMO-STAP suffered from severe performance loss. When *L* was larger than 10, EA-MIMO-STAP had almost no performance loss. The proposed EA-MIMO-STAP performed better than RD-MIMO-STAP and FD-MIMO-STAP in these cases; this verified that EA-MIMO-STAP is robust to clutter rank mismatch.

**Figure 11:**
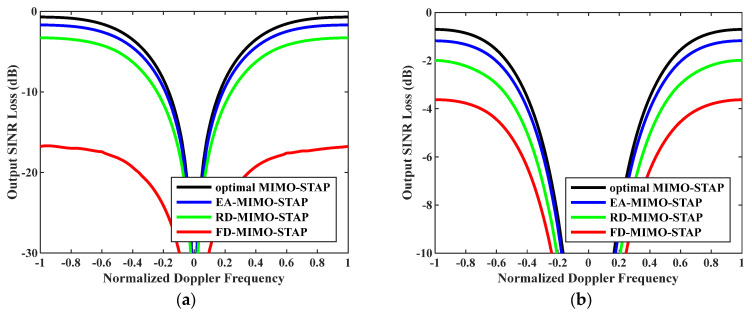
Output SINR losses versus normalized Doppler frequencies with V=300 m/s and $\sigma_{T}=0.144\;m$: (**a**) D=EMK and (**b**) D=2EMK.

Ninthly, output SINR losses versus normalized Doppler frequencies with increasing clutter rank are given in Figure 11. In the experiments, the numbers of snapshots were D=EMK and D=2EMK. The platform speed was configured to V=300 m/s as indicated, and the transmit antenna element spacing was set to $\sigma_{T}=0.144\;m$. In this case, as indicated by reference [47], an increase in speed led to a corresponding rise in the clutter rank. When this figure is compared with Figure 3, it becomes evident that the performance of all algorithms experienced a certain degree of degradation. Nevertheless, the proposed EA-MIMO-STAP algorithm continued to demonstrate outstanding performance. In Figure 11a, it can be observed that the output SINR loss incurred by the EA-MIMO-STAP algorithm dropped by roughly 15 dB and 1 dB, respectively, when compared to that incurred by the FD-MIMO-STAP algorithm and the RD-MIMO-STAP approach. In Figure 11b, while the output SINR loss of the EA-MIMO-STAP algorithm showed a decrease relative to that in Figure 11a, the EA-MIMO-STAP algorithm still outperformed the others, maintaining its superior performance.

**Figure 12:**
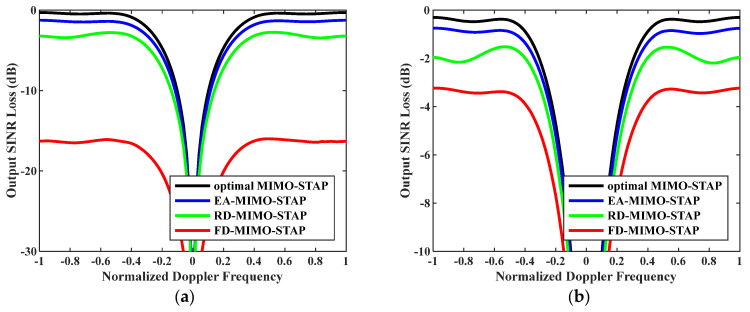
Output SINR losses versus normalized Doppler frequencies with V=150 m/s and $\sigma_{T}=0.288\;m$: (**a**) D=EMK and (**b**) D=2EMK.

Tenthly, Figure 12 presents the output SINR losses versus normalized Doppler frequencies with speed V=150 m/s and transmit antenna element spacing $\sigma_{T}=0.288\;m$. In the experiment, the numbers of snapshots were set to D=EMK and D=2EMK. According to reference [47], the clutter rank exhibits an upward trend as the ratio between the transmit and receive element spacings increases. When juxtaposed with the results in Figure 4, this figure shows that the performance of all algorithms experienced a decline. Nevertheless, the EA-MIMO-STAP algorithm continued to outperform both the RD-MIMO-STAP and FD-MIMO-STAP algorithms. Specifically, when compared to those for the conventional FD-MIMO-STAP approach, the SINR losses incurred by the EA-MIMO-STAP algorithm under D=EMK and D=2EMK decreased by approximately 16 dB and 3 dB, respectively.

**Figure 13:**
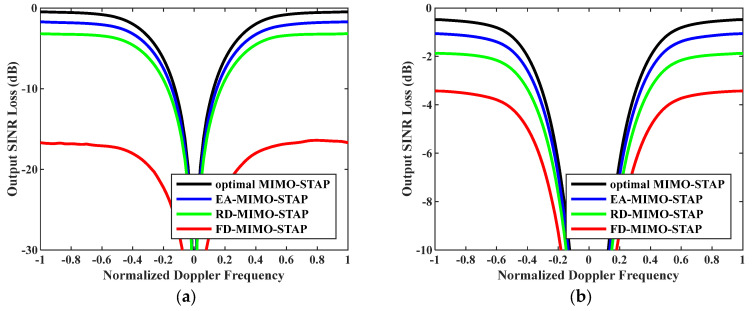
Output SINR losses versus normalized Doppler frequencies with V=300 m/s and $\sigma_{T}=0.288\;m$: (**a**) D=EMK and (**b**) D=2EMK.

Figure 13 gives output SINR losses versus normalized Doppler frequencies for speed V=300 m/s and transmit antenna element spacing $\sigma_{T}=0.288\;m$. In the experiment, the numbers of snapshots were set to D=EMK and D=2EMK. As with Figure 11 and Figure 12, all the algorithms’ performance declined. However, EA-MIMO-STAP still performed better than RD-MIMO-STAP and FD-MIMO-STAP. Specifically, in comparison with those for FD-MIMO-STAP, the SINR losses for EA-MIMO-STAP with D=EMK and D=2EMK decreased by about 17 dB and 2.5 dB, respectively.

**Figure 14:**
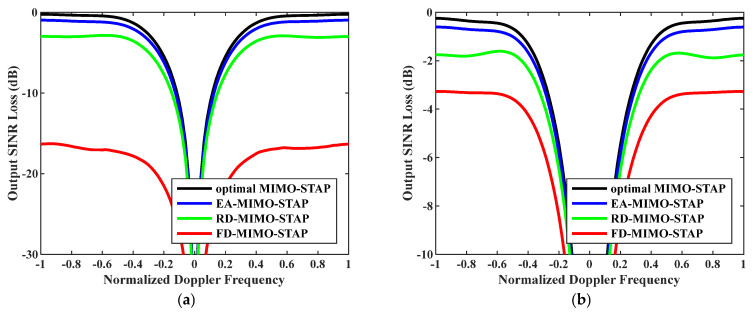

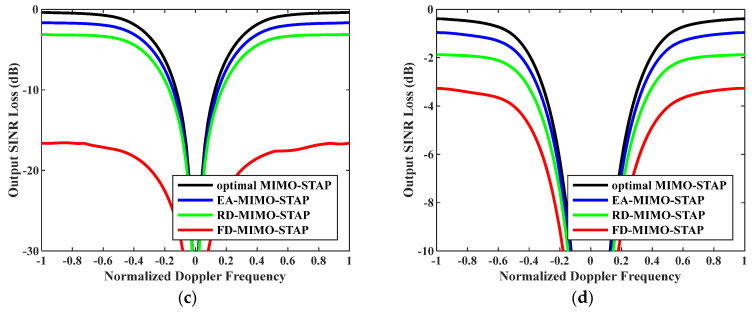
Output SINR losses versus normalized Doppler frequencies: (**a**) V=150 m/s, $\sigma_{T}=0.144\;m$, D=EMK; (**b**) V=150 m/s, $\sigma_{T}=0.144\;m$, D=2EMK; (**c**) V=300 m/s, $\sigma_{T}=0.288\;m$, D=EMK; and (**d**) V=300 m/s, $\sigma_{T}=0.288\;m$, D=2EMK.

In real-world scenarios, fluctuations in temperature and humidity often lead to inconsistencies in the gains and phases across receive arrays, a phenomenon typically characterized as array gain/phase errors, as detailed in reference [51]. These errors induce decorrelation among the transmit and receive channels, thereby degrading STAP performance. To assess the robustness of the EA-MIMO-STAP algorithm under such conditions, the experiment depicted in Figure 14 incorporated a 5% array gain/phase error into the clutter data. In the experiment, V=150 m/s and $\sigma_{T}=0.144\;m$, V=300 m/s and $\sigma_{T}=0.288\;m$, and D=EMK and D=2EMK were set. Compared to that shown in Figure 4 and Figure 12, the performance appeared to decline. We found that, for D=EMK, the proposed EA-MIMO-STAP algorithm demonstrated a substantial enhancement in performance when compared to both the RD-MIMO-STAP and FD-MIMO-STAP algorithms. For D=2EMK, while FD-MIMO-STAP, RD-MIMO-STAP, and EA-MIMO-STAP exhibited comparable performance levels, the EA-MIMO-STAP algorithm nonetheless stood out as the superior performer among them.

**Figure 15:**
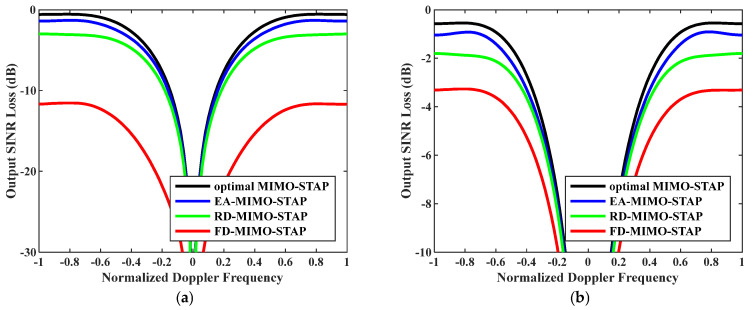

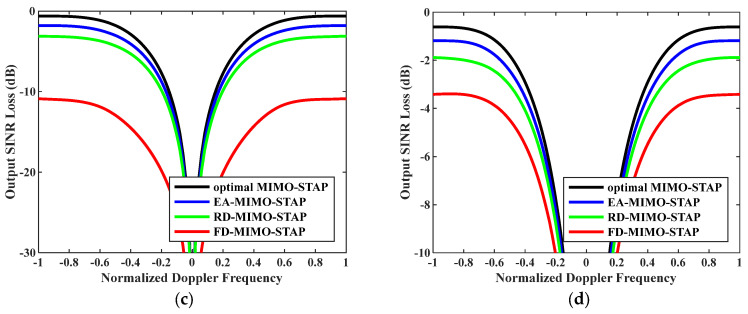
Output SINR losses versus normalized Doppler frequencies: (**a**) V=150 m/s, $\sigma_{T}=0.144\;m$, D=EMK; (**b**) V=150 m/s, $\sigma_{T}=0.144\;m$, D=2EMK; (**c**) V=300 m/s, $\sigma_{T}=0.288\;m$, D=EMK; and (**d**) V=300 m/s, $\sigma_{T}=0.288\;m$, D=2EMK.

In practice, internal clutter motion commonly has a significant effect on STAP performance because of wind, etc. This leads to decorrelation among the Doppler channels, degrading STAP performance [52]. To evaluate the robustness of the EA-MIMO-STAP algorithm under such conditions, the experiment depicted in Figure 15 incorporated a 1% internal clutter motion into the clutter generation. In the experiment, V=150 m/s and $\sigma_{T}=0.144\;m$, V=300 m/s and $\sigma_{T}=0.288\;m$, and D=EMK and D=2EMK were set. Compared to that shown in Figure 4 and Figure 12, the STAP performance appeared to decrease. Moreover, it was found that, regardless of the number of snapshots, the proposed EA-MIMO-STAP algorithm exhibited an outstanding and substantial enhancement in performance when benchmarked against both the RD-MIMO-STAP and FD-MIMO-STAP algorithms.

## 5. Conclusions

This work proposed a superior MIMO-STAP method with eigenvalue adjustment to enhance clutter rejection in airborne radar under limited snapshots. Leveraging the insights gleaned from the spiked framework, the proposed method precisely computes the inversion of the CPNCM estimate. This is achieved by fine-tuning the sample eigenvalues and preserving the fixed eigenvectors. In particular, noise eigenvalues are meticulously adjusted to align with the variance level of noise, whereas clutter eigenvalues are optimized based on sample eigenvalues of clutter through the minimization of radar output power. Owing to this refined CPNCM estimation technique, the proposed EA-MIMO-STAP algorithm not only markedly surpasses FD-MIMO-STAP and classical RD-MIMO-STAP in scenarios where the quantity of snapshots falls short of meeting the RMB rule but also maintains superior performance even when the RMB rule is fully satisfied.

## Figures and Tables

**Table 1:** Parameter settings.

Parameter	Value	Unit
Altitude	8000	m
Wavelength	0.288	m
Transmit antenna number	4	/
Receive antenna number	4	/
Receive antenna spacing	0.144	m
Pulse number	4	/
Pulse repetition frequency	2000	Hz
Noise power	1	W
CNR	30	dB

## Data Availability

The original contributions presented in this study are included in the article. Further inquiries can be directed to the corresponding author.
